# Prediction of Zn_2_(V, Nb, Ta)N_3_ Monolayers for Optoelectronic Applications

**DOI:** 10.1021/acs.jpclett.3c03206

**Published:** 2023-12-05

**Authors:** Andrey A. Kistanov, Svetlana V. Ustiuzhanina, Maryia S. Baranava, Dzmitry Ch. Hvazdouski, Stepan A. Shcherbinin, Oleg V. Prezhdo

**Affiliations:** † The Laboratory of Metals and Alloys Under Extreme Impacts, Ufa University of Science and Technology, Ufa 450076, Russia; ‡ Institute for Metals Superplasticity Problems, Russian Academy of Sciences, Ufa 450001, Russia; § Belarusian State University of Informatics and Radio Electronics, Minsk 22013, Belarus; ∥ Peter the Great Saint Petersburg Polytechnical University, Saint Petersburg 195251, Russia; ⊥ Institute for Problems in Mechanical Engineering RAS, Saint Petersburg 199178, Russia; # Department of Chemistry, University of Southern California, Los Angeles, California 90089, United States

## Abstract

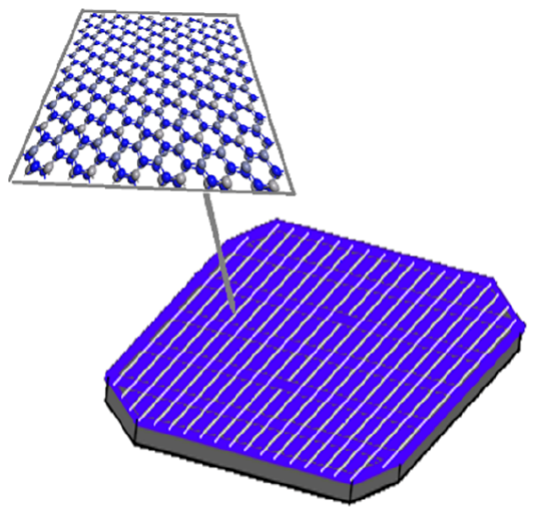

A new family of ternary nitride materials, Zn_2_(V, Nb, Ta)N_3_ monolayers, is predicted. A fabrication mechanism of the Zn_2_(V, Nb, Ta)N_3_ monolayers is proposed based on the chemical vapor deposition approach used for their bulk counterparts. The calculations show that these monolayers are thermodynamically and environmentally stable and that the Zn_2_VN_3_ monolayer is the most stable and the easiest to synthesize. The Zn_2_VN_3_ monolayer also has the highest strength and elasticity. The Zn_2_(V, Nb, Ta)N_3_ monolayers are semiconductors with nearly equal direct and indirect band gaps. Considering optoelectronic properties, the predicted monolayers are transparent to the visible light and provide shielding in the ultraviolet region. Thus, the predicted Zn_2_(V, Nb, Ta)N_3_ monolayers are promising for applications in LED devices and as blocking layers in tandem solar cells.

Computational modeling of materials’ properties can significantly facilitate experimental synthesis and discovery of materials with desired characteristics for specific applications. First-principles density functional theory (DFT) calculations facilitated discovery of a large number of novel nanostructures.^1−3^ For instance, the structure and some properties of two two-dimensional materials, arsenene and antimonene, have been predicted for the first time.^2^ The theoretical study motivated the corresponding synthetic efforts. Computational investigations also contributed to discovery of a stable borophene structure, which could not be synthesized for a long time due to its high polymorphism.^1,3^ The growing effectiveness of computational modeling and technical progress in the synthesis methods, make prediction of more complex nanostructures possible.^4−8^ DFT calculations have detected an exotic low-dimensional structure, a thin film of NaCl on the (110) surface of diamond, that was crystallized in an experiment based on the theoretical guidelines.^9^ Metastable iron disulfide FeS_2_ resulting from the full deintercalation of Li in Li_2_FeS_2_ has been elucidated via first-principles calculations accounting for experimental observations.^10^ Five new superhard W–Mo–B compounds have been predicted at different temperatures.^11,12^ The potential of ternary nitrides has been recently demonstrated in a comprehensive computational study^13^ through the prediction of hundreds of new stable and metastable ternary metal nitrides. Zn-based ternary nitride compounds have been characterized theoretically before their experimental realization.^14,15^ These examples demonstrate that computational methods can be effectively implemented to reduce experimental efforts in the study and characterization of complex structures.

Computational screening with a structure characterization using DFT calculations and the following physical vapor deposition synthesis of the Zn_2_VN_3_ thin film has been proposed recently by Zhuk et al.^16^ The optoelectronic properties of Zn_2_VN_3_ thin films, including a wide band gap of 2.35 eV, high charge carrier concentration of ∼10^17^ cm^–3^, and Hall mobility of 80 cm^2^/(V·s), make it a promising candidate for hole-selective contacts and hole transport layer applications in solar cells. Importantly, following the prediction and synthesis of Zn_2_VN_3_ thin films, the existence of the Zn_2_VN_3_ monolayer and characterization of its functional properties has been shown using DFT simulations.^15^ There are several computational DFT-based studies that have predicted stability of a group of semiconducting ternary nitride compounds such as Zn_2_NbN_3_ and Zn_2_TaN_3_.^17,18^ However, the experimental verification of these results has not been provided until another recent prediction has been made, accompanied by a subsequent synthesis of a Zn_2_NbN_3_ thin film.^19^ The combined experimental and modeling studies have demonstrated that Zn_2_NbN_3_ possess optoelectronic properties making it promising for design of epitaxial heterostructures with low strain using other ternary nitride compounds such as Mg_2_SbN_3_.^20^ Further experiments conducted by Zhuk et al.^16^ have verified formation of the new single-phase Zn_2_TaN_3_ prepared via reactive radio frequency cosputtering of Zn and Ta targets. It has been shown that Zn_2_TaN_3_ is a semiconductor possessing a wide band gap of 2.55 eV and a high resistivity of >10^5^ Ω·cm. The demonstrated stabilization of sputter-deposited Zn-based ternary nitride thin films at low synthesis temperatures and their chemical stability suggest new ways for fabrication of tandem perovskite-Si solar cells, in which the top layer with a high diffusion barrier is used to protect the Si-bottom cell.^21^ It is worth to note that the synthesis of the Zn-based ternary nitride compounds is challenging to due to their lower melting point and, as a consequence, a higher vapor pressure of zinc and zinc nitride compared to other metals.^19,22^

Limitations, related to Auger recombination, limit the theoretical performance efficiency of Si-based solar cells to 29.56%.^23^ One of the most promising approaches to overcoming this efficiency barrier involves stacking a wide band gap absorber layer on top of the Si layer to make a tandem solar cell. The wide band gap top layer will absorb the high-energy photons with reduced thermalization losses, while the narrow band gap Si bottom layer will absorb the low-energy photons of the solar spectrum.^24^ Ternary nitrides occupy their own niche in the tandem solar cell market, and the number of new functional ternary nitrides is increasing yearly. For instance, ZnGeN_2_ thin films have been implemented in the fabrication of light emitting diodes.^25^ A ZnSnN_2_ thin film has been utilized as an absorbing material is solar cells, with the ZnSnN_2_/*p*-CuCrO_2_ solar cell exhibiting an efficiency exceeding 22%.^26^ A ITO/TiZnN_2_/Si photodetector has been developed generating a photocurrent with a low light intensity and insensitive to humidity.^27^ It has also been shown that Zn_2_V_(1–*x*)_Nb_*x*_N_3_ alloys possess comparably small bowing parameter that is beneficial for materials used in light-emitting diodes.^28^

Another important question regarding the performance of tandem solar cells is related to the environmental stability of the top layer based on ternary nitrides. The stability or lifetime is a key merit to gauge the technical feasibility for commercialization of tandem solar cells. The current market of commercialized solar cells is mainly based of silicon due to a long lifetime and a good module efficiency of 21%.^29^ The lifetime of solar cells is affected by many factors, including exposure to moisture and oxygen.^29,30^ The lifetime energy yield and economic viability of ternary nitride/silicon tandem solar cells strongly depend on the degradation rates of ternary nitride cells.^31^

In this work, two novel Zn_2_NbN_3_ and Zn_2_TaN_3_ monolayers are reported for the first time, following recent works on the synthesis of Zn_2_(V, Nb, Ta)N_3_ thin films and the prediction of the Zn_2_VN_3_ monolayer. The structural characteristics and electronic properties of the monolayers are investigated by DFT calculations. Further, a systematic study of the structural integrity and resistance of the Zn_2_(V, Nb, Ta)N_3_ material family under chemically aggressive conditions, including exposure to oxygen and humidity, is conducted.

The model of the two novel members of the II_2_-V-N_3_ materials family, the Zn_2_NbN_3_ and Zn_2_TaN_3_ monolayers, is designed based on the Zn_2_VN_3_ monolayer structure obtained in the previous work,^15^Figures 1a-c show the optimized unit cells of the Zn_2_(V, Nb, Ta)N_3_ monolayers. Similar to the Zn_2_VN_3_ monolayer, the Zn_2_NbN_3_ and Zn_2_TaN_3_ monolayers stabilize in an orthorhombic lattice with space group 36 C*mc*2_1_. The lattice parameters are *a* = *b* = 5.77 Å and *a* = *b* = 5.78 Å, respectively (see cif files in the Supporting Information). The electron density for the Zn_2_(V, Nb, Ta)N_3_ monolayers reflected by the electronic localization function (ELF)^32^ with the isosurface value of 0.25 is incorporated in Figure 1. As the electron localization basin is spherical and completely migrates to the respective cores of the Zn and V(Nb, Ta) atoms, an ionic type of bonding in the Zn_2_(V, Nb, Ta)N_3_ monolayers is suggested.^33,34^ This is in line with the known rule that if the difference in the electronegativities of a bonded metal and nonmetal is above 1.5, a compound is expected to be ionic. The difference in the electronegativities of N (3.04), Zn (1.65), V (1.63), Nb (1.60), and Ta (1.50) is close to 1.5. Hence, the Zn_2_(V, Nb, Ta)N_3_ monolayers possesses ionic/ionocovalent bonds. Such a strong bonding in the Zn_2_(V, Nb, Ta)N_3_ monolayers suggest stability and high structural integrity against the intrinsic defects formation.^35^

**Figure 1:**
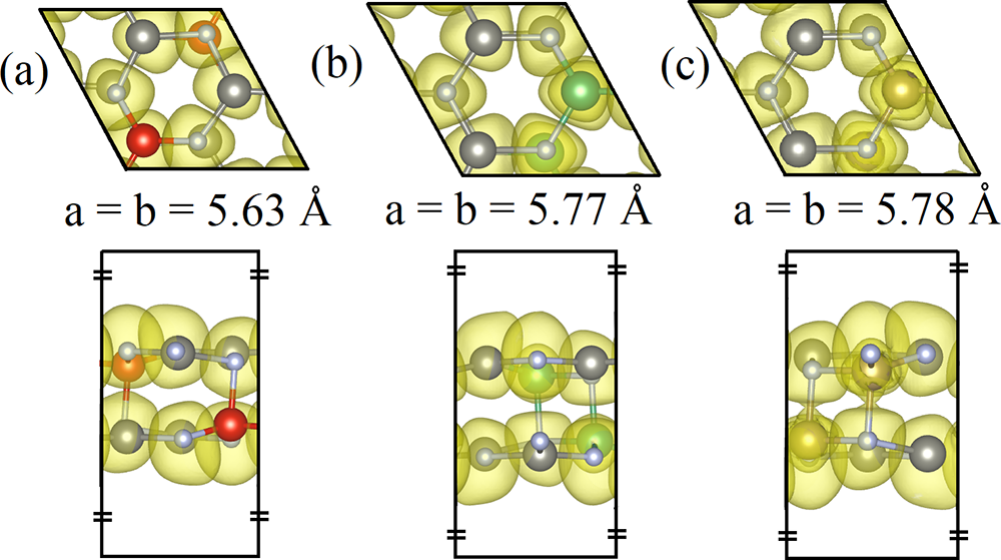
(a) Zn_2_VN_3_, (b) Zn_2_NbN_3_, and (c) Zn_2_TaN_3_ monolayer unit cells combined with ELF. The Zn, V, Nb, Ta, and N atoms are colored gray, red, green, golden, and violet, respectively.

To confirm the stability of the Zn_2_Nb(Ta)N_3_ monolayers, their phonon spectra are calculated. According to Figure S1, the phonon dispersion spectra exhibit no regions with imaginary (negative) frequencies, implying that the Zn_2_Nb(Ta)N_3_ monolayers are dynamically stable. To confirm the dynamical stability of the Zn_2_(V, Nb, Ta)N_3_ monolayers, ab initio molecular dynamics (AIMD) simulations are used to demonstrate that exist no structural changes that lower the system energy.^36^ As a solar panel temperature generally ranges between ∼280 °C and ∼310 °C during which solar cells produce maximum efficiency,^37,38^ AIMD simulations are conducted at temperatures of 280 and 300 K to evaluate the working temperature conditions of the considered monolayers. The Zn_2_(V, Nb, Ta)N_3_ monolayers remain stable after at least 5 ps (Figures S2–S4) as there neither structural changes nor energy and temperature fluctuations are observed.

Mechanical stability is another important characteristic for materials used in optoelectronic and photovoltaic nanodevices.^39,40^ According to the established rules,^41^ orthorhombic two-dimensional systems are mechanically stable if the following criteria are satisfied:
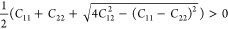
1
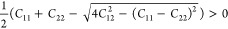
2
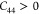
3

The calculated elastic constants *C*_ij_ for the Zn_2_(V, Nb, Ta)N_3_ monolayers are collected in Table S1. The substitution of the values of the elastic constants from Table S1 to eq 1 shows that the criteria presented in eqs 1–3 are satisfied for the Zn_2_(V, Nb, Ta)N_3_ monolayers, which confirms their mechanical stability.

“Potentially exfoliable” 2D systems can be evaluated theoretically via the exfoliation energy Δ*E*_exf_ that should be limited to 200 meV/ Å^2^ (the details can be found in the Methods).^42^ The calculated Δ*E*_exf_ for the Zn_2_(V, Nb, Ta)N_3_ monolayers is 105 meV/Å^2^, 117 meV/Å^2^, and 125 meV/Å^2^, respectively, indicating that these monolayers can be exfoliated under certain conditions. According to the recently reported deposition approach, evaporation of Zn_3_N_2_ and VN in ionized nitrogen at a temperature of 390–490 K forms a Zn_2_VN_3_ thin according to the reaction Zn_3_N_2_ + VN → (N+) Zn_2_VN_3_ + Zn (evaporation).^16^ It has also been reported that 150 nm Zn–Nb–N thin films can be deposited by cosputtering from Zn and Nb targets in nitrogen plasma without intentional heating.^19^ Further, Zn_*x*_Ta_1–*x*_N thin films have been fabricated using reactive radio frequency cosputtering of Zn and Ta targets at a low temperature of approximately 70 to 150 °C.^43^ In this work, AIMD simulations are conducted to simulate the formation of the Zn_2_(V, Nb, Ta)N_3_ monolayers, motivated by the experimental data available for thin films of these materials. As shown in Figure 2a, two targets are used for sputtering: Zn and V(Nb, Ta). Co-sputtering is conducted in ionized nitrogen and at the temperature of 150–180 °C. In AIMD simulations performed under the above-mentioned conditions it has been found that Zn_2_VN_3_ (Figure 2b), Zn_2_VN_3_ (Figure S5a), and Zn_2_VN_3_ (Figure S5b) hexagons with an extra (unevaporated) Zn atom can be formed at ∼180 °C within ∼4 ps. The process can be adjusted by controlling specific parameters, such as the Zn and Zn_3_N_2_ evaporation rates,^44^ the ionized nitrogen rate,^16^ and the synthesis temperatures.^19^

**Figure 2:**
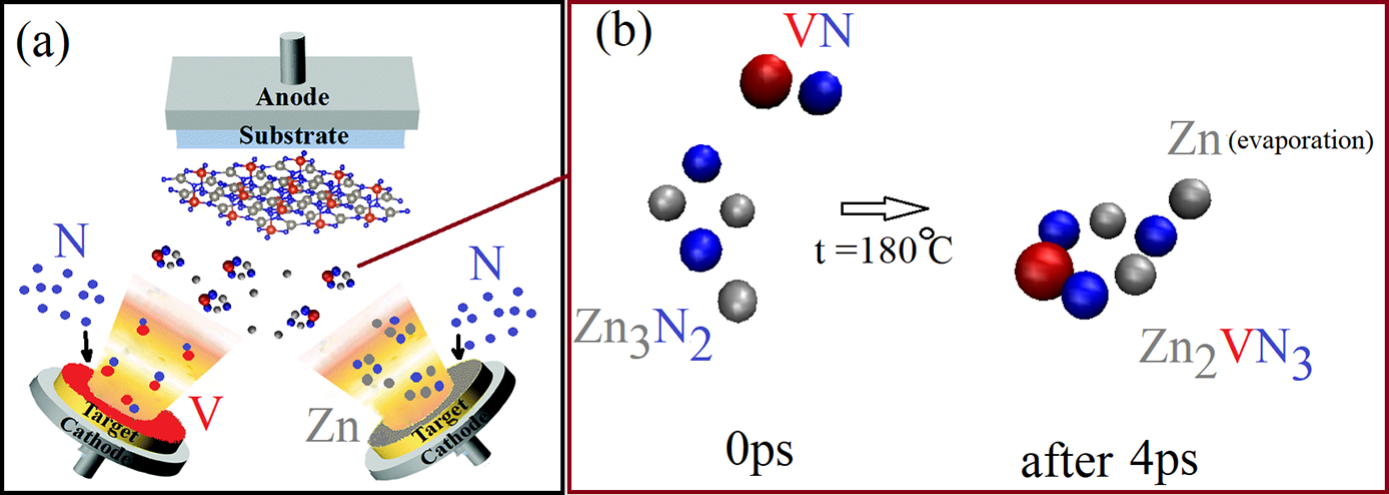
(a) Schematic of the Zn_2_(V, Nb, Ta)N_3_ monolayer synthesis by magnetron sputtering. (b) 180 °C AIMD simulation of formation Zn_2_VN_3_ hexagons–building blocks of the Zn_2_VN_3_ monolayer.

The calculation of the X-ray diffraction (XRD) patterns^45^ of the bulk and monolayer Zn_2_(V, Nb, Ta)N_3_ is carried out to allow experimental identification of these structures. The XRD patterns and the unit cell of bulk Zn_2_(V, Nb, Ta)N_3_ are shown in Figures S6a–c. The Zn_2_(V, Nb, Ta)N_3_ XRD patterns calculated here are similar to the previously obtained experimental XRD patterns.^16,43^ The XRD patterns and the unit cell of the Zn_2_(V, Nb, Ta)N_3_ monolayers are shown in Figures S6d–f. As expected, the relative intensities of the main peaks vary for the monolayers and their thin films counterparts.^8^

Optoelectronic and mechanical properties of the Zn_2_(V, Nb, Ta)N_3_ monolayers are considered next. Figures 3a–c present the band structure of the Zn_2_(V, Nb, Ta)N_3_ monolayers obtained using the Heyd–Scuseria–Ernzerhof (HSE06) exchange-correlation functional. The Zn_2_VN_3_ monolayer (Figure 3a) has an indirect band gap of 2.75 eV and a direct band gap of 2.85 eV, as it has been reported previously.^15^ In turn, the Zn_2_NbN_3_ and Zn_2_TaN_3_ monolayers have an indirect band gap that is equal to the direct band gap. The band gap of the Zn_2_NbN_3_ monolayer is 3.38 eV (Figure 3b), while the band gap of the Zn_2_TaN_3_ monolayer is 3.33 eV (Figure 3c). Since the Zn_2_VN_3_ monolayer has an indirect band gap, photoexcitation of a photon at the band gap energy requires coupling to a phonon, which lowers its absorption coefficient and makes solar devices based on Zn_2_VN_3_ monolayers thicker. On the other hand, the indirect band gaps of the Zn_2_NbN_3_ and Zn_2_TaN_3_ monolayers are equal to the direct band gap. Therefore, light absorption is more efficient, and it should be possible to decrease the thickness of solar devices based on these materials. It is found that the Zn_2_(V, Nb, Ta)N_3_ monolayers exhibit *p*-type conductivity. The conduction band minimum (CBM) of the Zn_2_NbN_3_ and Zn_2_TaN_3_ monolayers is located at the S point, while the valence band maximum (VBM) is located at the *Y* point (for the indirect band gap). The partial density of states (PDOS) for the Zn_2_(V, Nb, Ta)N_3_ monolayer depicted in Figure S7 shows that the CBM is mainly formed by d states of V(Nb, Ta) atoms, while the VBM is formed by p states of N atoms. A strong CBM localization and band curvature can be seen in the band structure and PDOS plots, in particular, in the Zn_2_NbN_3_ and Zn_2_TaN_3_ monolayers. The CBM localization, found previously in the Zn_2_VN_3_ monolayer and observed here for the Zn_2_NbN_3_ and Zn_2_TaN_3_ monolayers, can be attributed to the cation disordering.^27^ The cation disordering is stronger in the Zn_2_NbN_3_ and Zn_2_TaN_3_ monolayers, which is the underlying reason for their unique band structure with equal direct and indirect band gaps.

**Figure 3:**
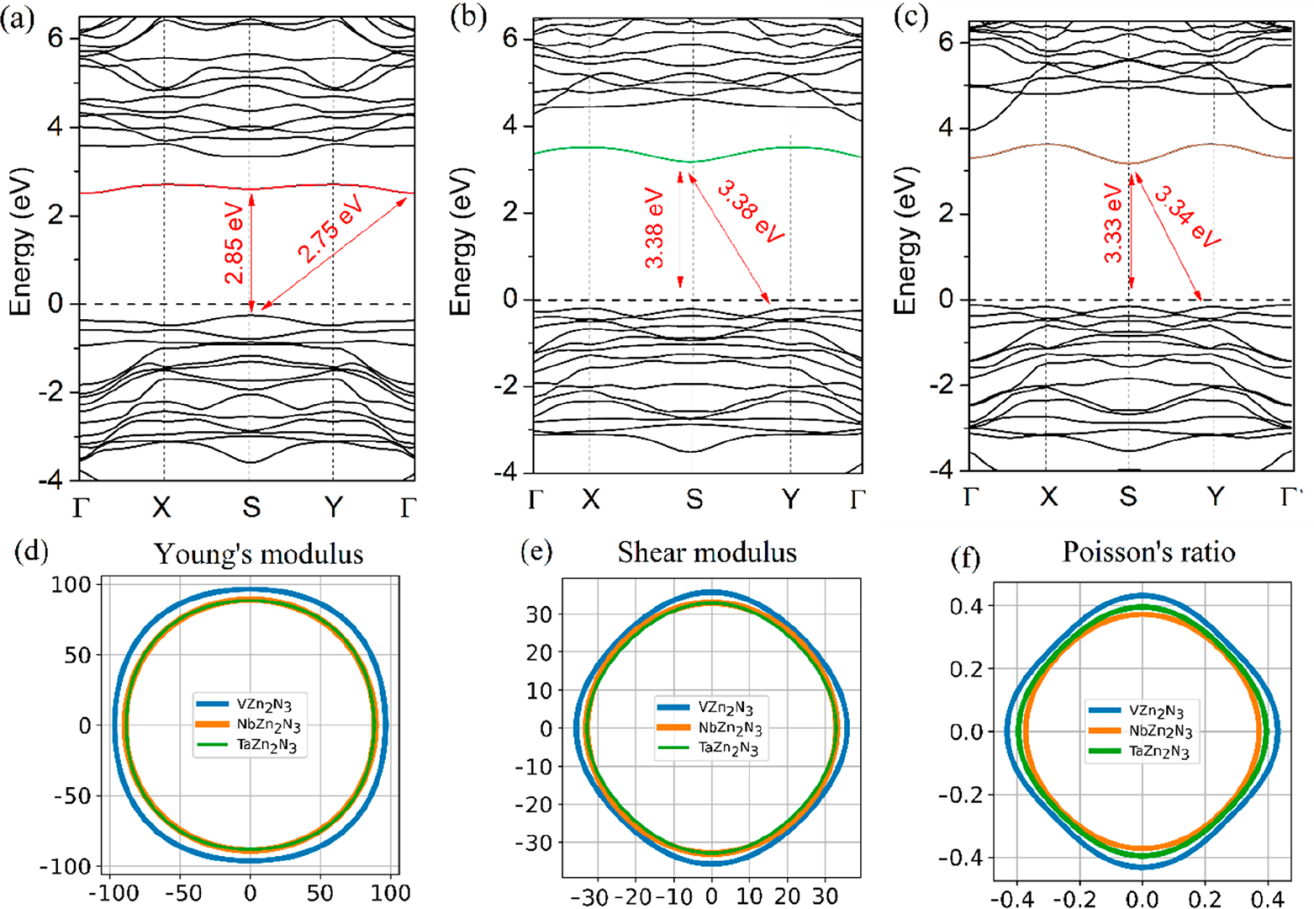
Band structure of (a) Zn_2_VN_3_, (b) Zn_2_NbN_3_, and (c) Zn_2_TaN_3_ monolayers calculated using the HSE approach. Spatial dependencies of (d) Young’s modulus (N/m), (e) shear modulus (N/m), and (f) Poisson’s ratio for the Zn_2_(V, Nb, Ta)N_3_ monolayers.

The calculated work functions (WFs) of the Zn_2_(V, Nb, Ta)N_3_ monolayers are shown in Figure S8, in comparison to other common 2D materials and bulk metals. The Zn_2_TaN_3_ monolayer has the highest WF value of 5.34 eV, comparable to those of bulk nickel^46^ and borophene monolayer,^47^ while lower than that of bulk platinum.^46^ The Zn_2_VN_3_ and Zn_2_NbN_3_ monolayers have only slightly lower WF values of 5.27 and 5.31 eV, respectively. According to the orbital resolved plots for the Zn_2_(V, Nb, Ta)N_3_ monolayers shown in Figure S9, the atomic states around the Fermi level consist of the out-of-plane and the in-plane hybridized states. Thus, a significant amount of energy is required for ionization of the Zn_2_(V, Nb, Ta)N_3_ monolayers, which is the underlying reason for their high WF values.

To gain insights into mechanical properties of the Zn_2_(V, Nb, Ta)N_3_ monolayers, the spatial dependence of Young’s modulus, the shear modulus, and Poisson’s ratio are calculated, as shown in Figures 3d–f. Young’s moduli (Figure 3d) of the Zn_2_VN_3_, Zn_2_NbN_3_, and Zn_2_TaN_3_ monolayers are nearly isotropic and are equal to 96.4, 89.0, and 88.3 N/m, respectively. In turn, the shear moduli (Figure 3e) and Poisson’s ratios (Figure 3f) of the Zn_2_V(Nb,Ta)N_3_ monolayers possess slight isotropy. The highest values of shear moduli are 35.7 (Zn_2_VN_3_ monolayer), 33.0 (Zn_2_NbN_3_ monolayer), and 32.8 N/m (Zn_2_TaN_3_ monolayer). The highest values of Poisson’s ratios are 0.43 (Zn_2_VN_3_ monolayer), 0.37 (Zn_2_NbN_3_ monolayer), and 0.40 (Zn_2_TaN_3_ monolayer). It should be noted that the calculated mechanical properties are defined for the monolayer and need to be rescaled by dividing the value of the calculated Young’s modulus or shear modulus by the thickness of the quasi-2D systems or thin films along the out-of-plane direction.^48^ Considering the bulk counterparts of the considered monolayers, the shear modulus and Poisson’s ratio are 61 GPa and 0.32 for bulk Zn_2_VN_3_, and 53 GPa and 0.33 for bulk Zn_2_NbN_3_.^49^ Thus, the stiffness decreases slightly from bulk Zn_2_VN_3_ to bulk Zn_2_NbN_3_, while the elasticity slightly increases. For the monolayers, the stiffness and the elasticity decrease slightly from the Zn_2_VN_3_ monolayer to the Zn_2_TaN_3_ monolayer.

The calculated real and imaginary parts of the complex dielectric function for the Zn_2_(V, Nb, Ta)N_3_ monolayers are presented in Figure S10. The static dielectric functions (at 0 eV in Figure S10a–c), representing the dielectric response of the materials to a static electric field, are 2.41, 2.36, and 2.41 for the Zn_2_VN_3_, Zn_2_NbN_3_, and Zn_2_TaN_3_ monolayers, respectively. The maxima of the real parts of the dielectric functions for the Zn_2_VN_3_, Zn_2_NbN_3_, and Zn_2_TaN_3_ monolayers is found to be 2.92 at 2.14 eV (Figure S10a), 2.62 at 3.07 eV (Figure S10b), and 3.14 at 3.74 eV (Figure S10c), respectively. The real parts of the dielectric functions are positive, indicating the material’s ability to support propagation of electromagnetic waves. The imaginary parts of the dielectric functions in Figures S10d–f show that the Zn_2_(V, Nb, Ta)N_3_ monolayers absorb light in the visible and ultraviolet regions. The frequency-dependent absorbance *A*(*ω*) can be calculated from the real ε_*re*_(*ω*) and imaginary ε_*im*_(*ω*) parts of the dielectric function *ε*(*ω*) using optical conductivity.^50^

The calculated linear optical spectra of the Zn_2_(V, Nb, Ta)N_3_ monolayers are shown in Figure 4. The Zn_2_(V, Nb, Ta)N_3_ monolayers reflect light in the far-infrared and infrared regions from 0.1 to 1.65 eV. The Zn_2_VN_3_, Zn_2_NbN_3_, and Zn_2_TaN_3_ monolayers absorb light in the visible range from 1.65 to 3.26 eV for photons with energies higher than 1.68, 2.21, and 2.16 eV, respectively (Figure 4d–f). The maximum absorption value of 16.06% for the Zn_2_VN_3_ monolayer is found in the middle ultraviolet region in the range from 4.13 to 6.20 eV for photons with energies of 4.98 eV. The maximum absorption value reaches 17.46% (photon energy is 6.21 eV) and 17.72% (photon energy is 6.26 eV) for the Zn_2_NbN_3_ and Zn_2_TaN_3_ monolayers, in the far ultraviolet region in the range from 6.20 to 10.16 eV.

**Figure 4:**
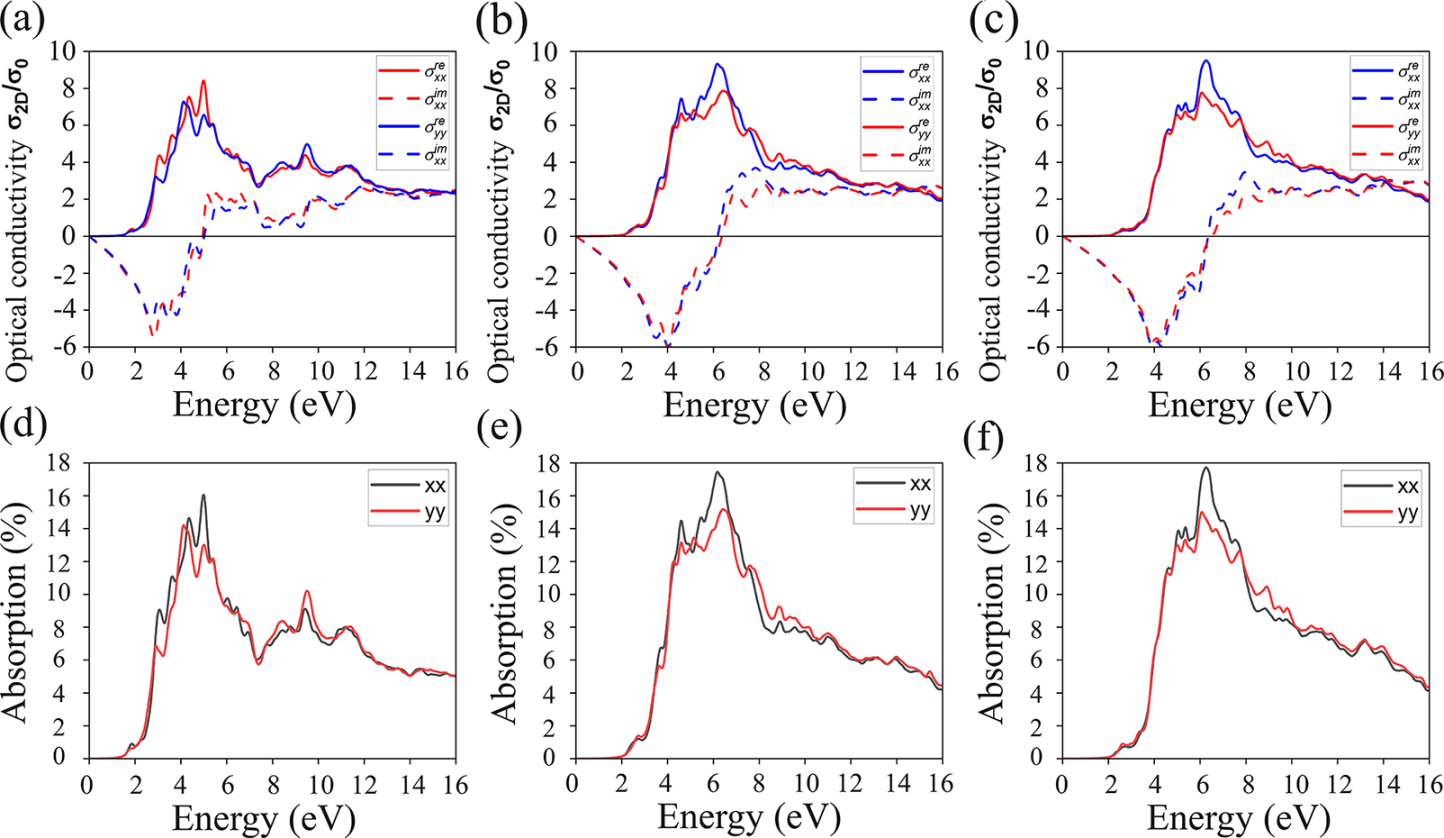
Real (solid line) and imaginary (dash line) parts of the frequency dependent optical conductivity for the (a) Zn_2_VN_3_, (b) Zn_2_NbN_3_, and (c) Zn_2_TaN_3_ monolayers in the *xx* (red line) and *yy* (blue line) directions. Absorption spectra *A*(ω) of the (d) Zn_2_VN_3_, (e) Zn_2_NbN_3_, and (f) Zn_2_TaN_3_ monolayers in *xx* (black line) and *yy* (red line) directions.

Applications of 2D materials depend strongly on their structural integrity under environmental conditions.^51,52^ Previously, the Zn_2_VN_3_ monolayer has shown excellent resistance to moisture.^15^ Here, the Zn_2_NbN_3_ and Zn_2_TaN_3_ monolayers are tested for exposure to H_2_O and O_2_ molecules. The energies of O_2_ adsorption, *E*_a_, on the Zn_2_NbN_3_ and Zn_2_TaN_3_ monolayers are −0.11 and −0.19 eV, respectively, while the corresponding H_2_O adsorption energies are −0.64 and −0.73 eV, respectively. Thus, the adsorption of H_2_O is more favorable compared to O_2_. The detailed pathways from the initial state (IS) through the transition state (TS) to the final state (FS) for the dissociative adsorption of the H_2_O and O_2_ molecules on the Zn_2_NbN_3_ and Zn_2_TaN_3_ monolayers are shown in Figure 5. The lowest energy configurations for H_2_O and O_2_ on the Zn_2_NbN_3_ and Zn_2_TaN_3_ monolayers corresponds to the IS. The calculated energy barriers *E*_b_ for H_2_O and O_2_ on Zn_2_NbN_3_ are 0.33 and 0.42 eV, respectively. The energy barriers for H_2_O and O_2_ on Zn_2_TaN_3_ are 0.21 and 0.27 eV, respectively. Thus, according to the obtained results, both H_2_O and O_2_ possess low *E*_b_. On the other hand, adsorption of H_2_O on the Zn_2_NbN_3_ and Zn_2_TaN_3_ monolayers is stronger than adsorption of O_2_.

**Figure 5:**
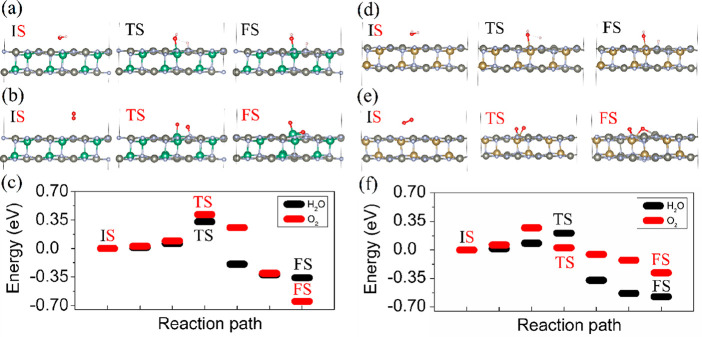
Atomic configurations corresponding to the dissociation process of H_2_O on (a) Zn_2_NbN_3_ and (d) Zn_2_TaN_3_ monolayers and O_2_ on (b) Zn_2_NbN_3_ and (e) Zn_2_TaN_3_ monolayers. (c) Energy profiles of the reaction pathway for H_2_O and O_2_ on (c) Zn_2_NbN_3_ and (f) Zn_2_TaN_3_ monolayers.

Based on the rate theory, the transition time for chemisorption of O_2_ on the Zn_2_NbN_3_ and Zn_2_TaN_3_ monolayers is estimated as follows:
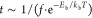
4where *E*_b_ is the energy barrier, *k*_b_ is the Boltzmann constant, *T* is temperature, and *f* is the attempt frequency. The latter is defined as *f* = *n*·*v*·*s*_d_, where *n* is the density of the O_2_ in air, *v* is speed, and *s*_d_ can be taken as the square of the lattice parameter. Hence, at the room temperature of 300 K, the time needed for O_2_ chemisorption on the Zn_2_NbN_3_ and Zn_2_TaN_3_ monolayers is 7 × 10^–1^ s and 7 × 10^–4^ s, respectively. The results suggest that a large amount of O_2_ molecules is able to chemisorb from air on both Zn_2_NbN_3_ and Zn_2_TaN_3_ monolayers at room temperature. It should be noted that H_2_O is crucial for the stability of 2D surfaces. OH-saturated surfaces exhibit higher stability against oxidation due to repulsion between the OH-groups in the saturated layer and the O_2_ molecules in the air.^53,54^

## Methods

Spin-polarized calculations within the DFT framework were performed using the Vienna Ab initio Simulation Package (VASP).^55^ The Perdew–Burke–Ernzerhof (PBE) functional^56^ under the generalized gradient approximation was used for the geometry optimization, the AIMD simulations and the mechanical properties calculations, while the electronic properties were obtained using the more accurate HSE06 hybrid exchange-correlation functional.^57^ The atomic force and total energy thresholds for the geometry optimization were 10^–4^ eV/Å and 10^–8^ eV, respectively. The plane-wave basis cutoff energy was set to 540 eV. Periodic boundary conditions were implemented in the in-plane transverse directions, while a vacuum space of 20 Å was introduced to the out-of-plane direction. The DFT-D3 dispersion correction was included to treat the van der Waals interaction.^58^

The phonon spectra were calculated in the framework of the density functional perturbation theory,^59^ as implemented in the Phonopy.^60^ 5 ps AIMD trajectories were generated using a 1.0 fs time step. The Nose–Hoover thermostat was used to control the temperature.^61^ The exfoliation energy, Δ*E*_exf_, was calculated as follows:^62^
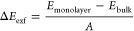
5where *E*_monolayer_ and *E*_bulk_ are the energies of the optimized monolayer and bulk Zn_2_ (V, Nb, Ta)N_3_, and *A* is the in-plane surface area according to the optimized bulk Zn_2_ (V, Nb, Ta)N_3_.

The climbing image–nudged elastic band method^63^ was used to obtain the energy profiles and reaction pathways of the H_2_O and O_2_ molecules on the surface. Directional dependencies of Young’s modulus, the shear modulus, and Poisson’s ratio were obtained using the stress–strain relation and were analyzed and plotted thought the ELATE software.^64^ Young’s modulus and Poisson’s ratio in the *x* and *y* directions and the shear modulus were calculated based on eqs 6–8, respectively.^41,65^
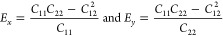
6
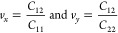
7
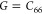
8

The simulated XRD plots were obtained with the VESTA software.^66^ For the XRD analysis, the incident wavelength λ of 1.542 Å corresponding to the Cu–Kα radiation was used.

The optical conductivity was calculated using the PBE functional based on the Maxwell equation:^67^
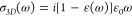
9where *ε*(*ω*) is the frequency-dependent complex dielectric function, ε_0_ is the permittivity of vacuum, and ω is the frequency of the incident wave.

As the dielectric function of materials depends on the thickness of the vacuum layer,^68^ in the case of monolayer,^69,70^eq 9 can be adjusted as follows:
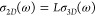
10Here *L* is the slab thickness in the simulation cell.

The normalized absorbance *A*(ω) was calculated as follows:
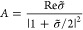
11where σ̃(ω) = σ_*2D*_(*ω*)/*ε*_0_*c* is the normalized conductivity, and *c* is the speed of light). Since only the interband contribution is considered, eq 11 is valid for both semiconducting and insulating 2D crystals.

## Supporting Information Available

Phonon dispersion curves of the Zn_2_(V, Nb, Ta)N_3_ monolayers (Figure S1), AIMD results for the Zn_2_(V, Nb, Ta)N_3_ monolayers (Figures S2–S4), calculated elastic constants for the Zn_2_(V, Nb, Ta)N_3_ monolayers (Table S1), AIMD results on the formation of Zn_2_NbN_3_, and Zn_2_TaN_3_ hexagons (Figure S5), XRD of thin film and monolayer of Zn_2_(V, Nb, Ta)N_3_ (Figure S6), PDOS of the Zn_2_(V, Nb, Ta)N_3_ monolayers (Figure S7), WF of the Zn_2_(V, Nb, Ta)N_3_ monolayers (Figure S8), orbital resolved DOS of the Zn_2_(V, Nb, Ta)N_3_ monolayers (Figure S9), and dielectric function of the Zn_2_(V, Nb, Ta)N_3_ monolayers (Figure S10) (PDF)
Zn_2_VN_3_ monolayer primitive cell (CIF)
Zn_2_NbN_3_ monolayer primitive cell (CIF)
Zn_2_TaN_3_ monolayer primitive cell (CIF)

## Supplementary Material

jz3c03206_si_001.pdf

jz3c03206_si_002.cif

jz3c03206_si_003.cif

jz3c03206_si_004.cif
