# Supplementary material for: Prediction of Zn2(V, Nb, Ta)N3 Monolayers for Optoelectronic Applications
Source: J Phys Chem Lett. 2023 Dec 5;14(49):11134–41. doi: 10.1021/acs.jpclett.3c03206 (PMC10726353; doi:10.1021/acs.jpclett.3c03206)
Supplement: Supplementary file 1 — jz3c03206_si_001.pdf [file jz3c03206_si_001.pdf]

Supporting Information for

Prediction of $Zn_2(V, Nb, Ta)N_3$ Monolayers for Optoelectronic Applications

Andrey A. Kistanov,[1,] Svetlana V. Ustiuzhanina,[2] Maryia S. Baranava,[3] Dzmitry Ch. Hvazdouski,[3] Stepan A. Shcherbinin,[4,5] Oleg V. Prezhdo[6]*

[1]The Laboratory of Metals and Alloys Under Extreme Impacts, Ufa University of Science and Technology, Ufa 450076, Russia

[2]Institute for Metals Superplasticity Problems, Russian Academy of Sciences, Ufa 450001, Russia

[3]Belarusian State University of Informatics and Radio Electronics, Minsk 22013, Belarus

[4]Peter the Great Saint Petersburg Polytechnical University, Saint Petersburg 195251, Russia

[5]Institute for Problems in Mechanical Engineering RAS, Saint Petersburg 199178, Russia

[6]Department of Chemistry, University of Southern California, Los Angeles, California 90089, United States

*Corresponding author: andrei.kistanov.ufa@gmail.com

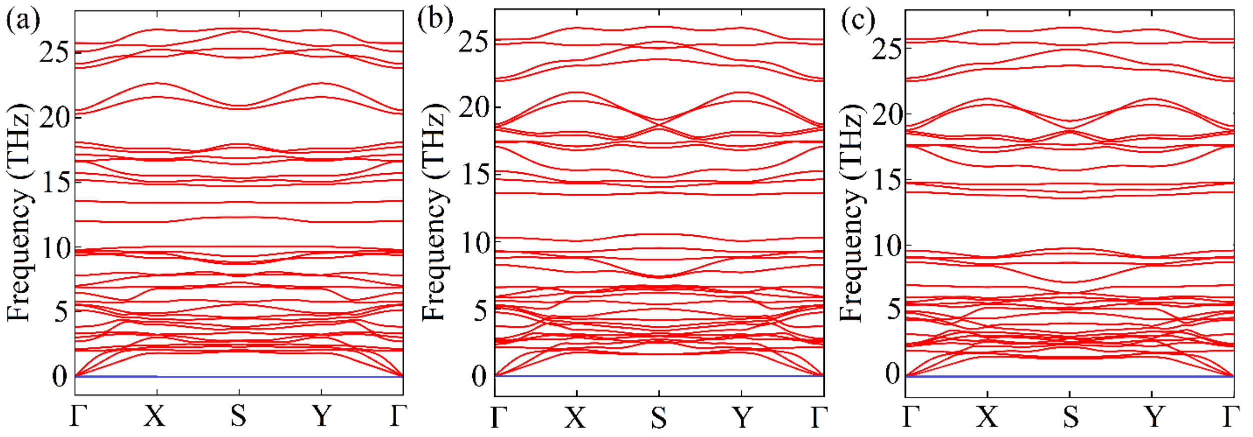

Figure S1. Phonon dispersion curves of the **(a)** Zn_2VN_3, **(b)** Zn_2NbN_3, and **(c)** Zn_2TaN_3 monolayers calculated along the high symmetry path of the Brillouin zone. The blue line shows the Fermi level.

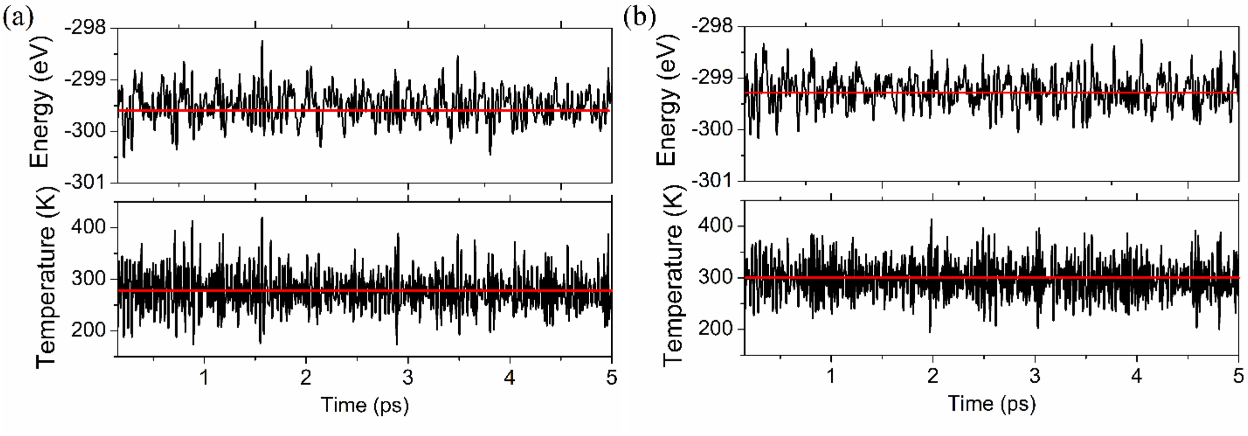

Figure S2. AIMD results at **(a)** 280 K and **(b)** 300 K on the energy fluctuation of the Zn_2VN_3 monolayer.

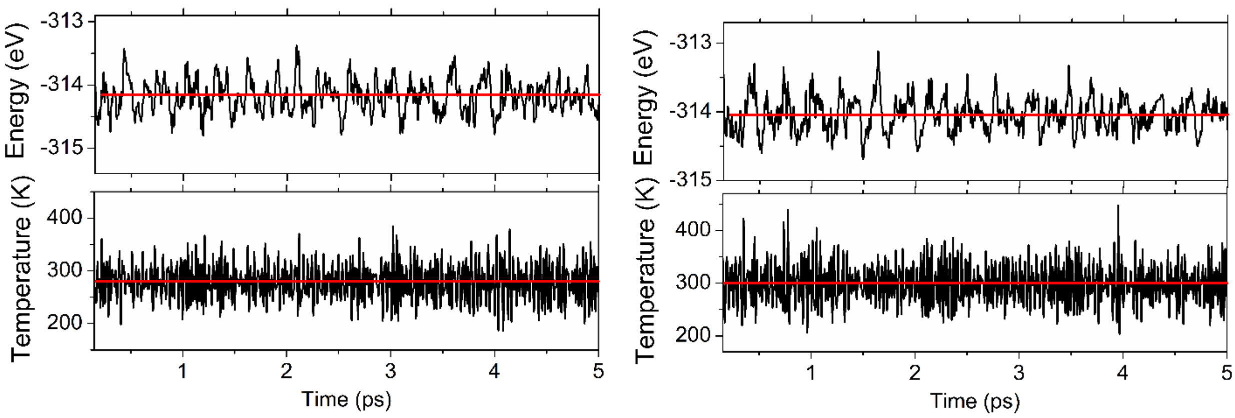

Figure S3. AIMD results at **(a)** 280 K and **(b)** 300 K on the energy fluctuation of the Zn_2NbN_3 monolayer.

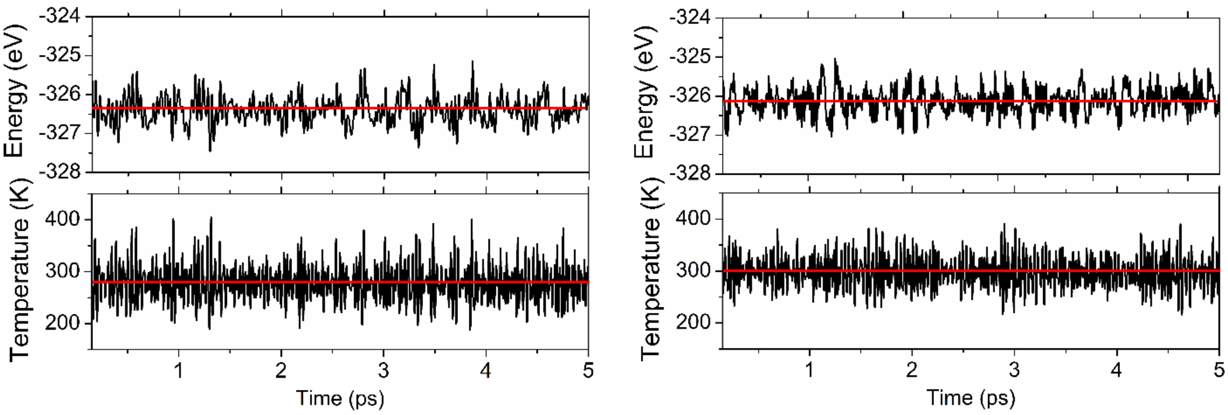

Figure S4. AIMD results at **(a)** 280 K and **(b)** 300 K on the energy and temperature fluctuation of the Zn_2TaN_3 monolayer.

Table S1. The calculated elastic constants C_{ij}.

	Zn_2VN_3 monolayer	Zn_2NbN_3 monolayer	Zn_2TaN_3 monolayer
C_{11}, N/m	421	370	380
C_{22}, N/m	421	370	380
C_{12}, N/m	182	138	151
C_{44}, N/m	126	117	119

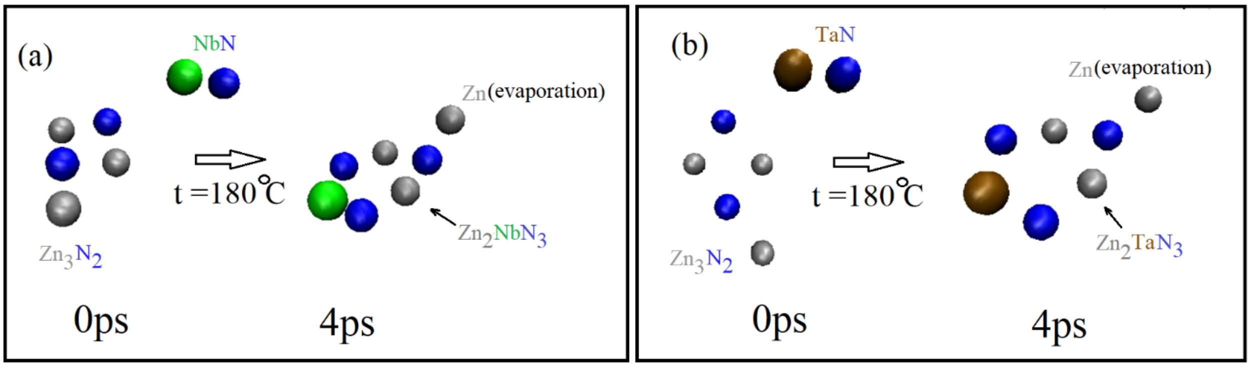

Figure S5. AIMD simulations of the **(a)** Zn_2NbN_3 and **(b)** Zn_2TaN_3 hexagons – building blocks of the Zn_2NbN_3 and Zn_2TaN_3 monolayer, respectively, - formation at the temperature of 180 ℃.

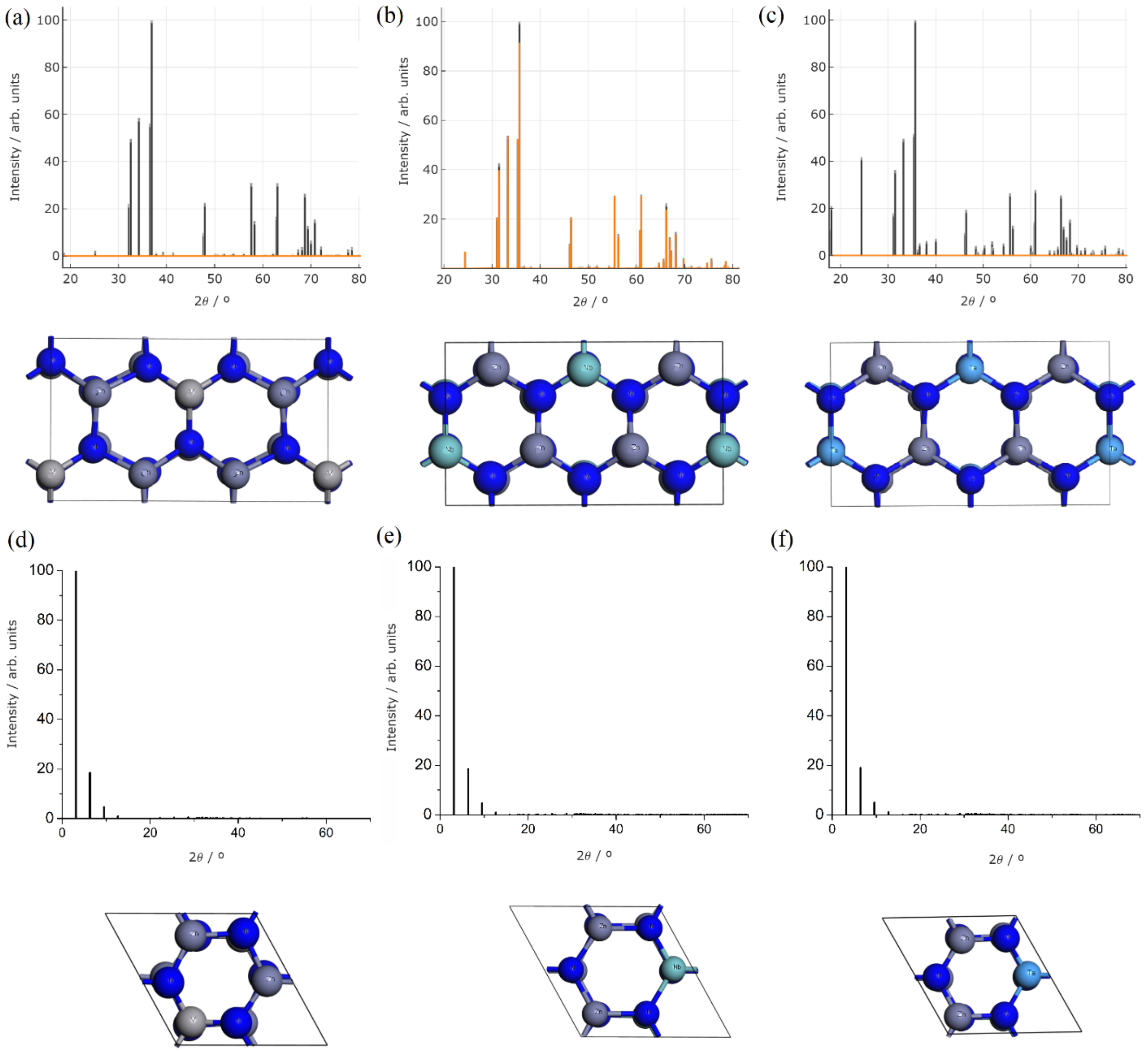

Figure S6. XRD of thin film and monolayer of **(a)** and **(d)** Zn_2VN_3, **(b)** and **(e)** Zn_2NbN_3, and **(c)** and **(f)** Zn_2TaN_3, respectively.

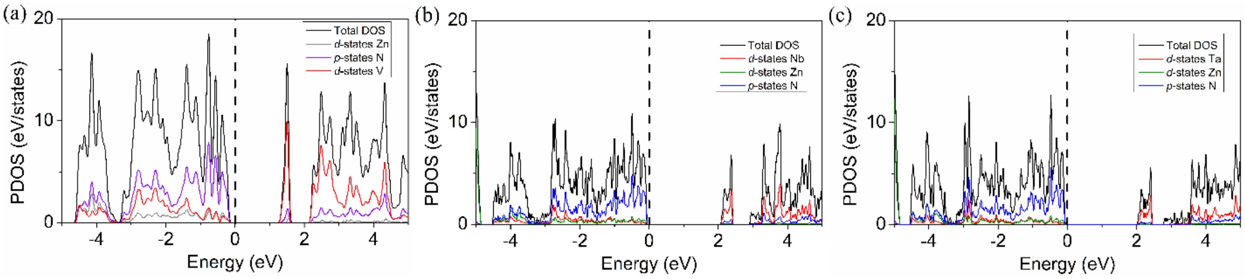

Figure S7. PDOS of the **(a)** Zn_2VN_3, **(b)** Zn_2NbN_3, and **(c)** Zn_2TaN_3 monolayers, obtained via the PBE GGA functional.

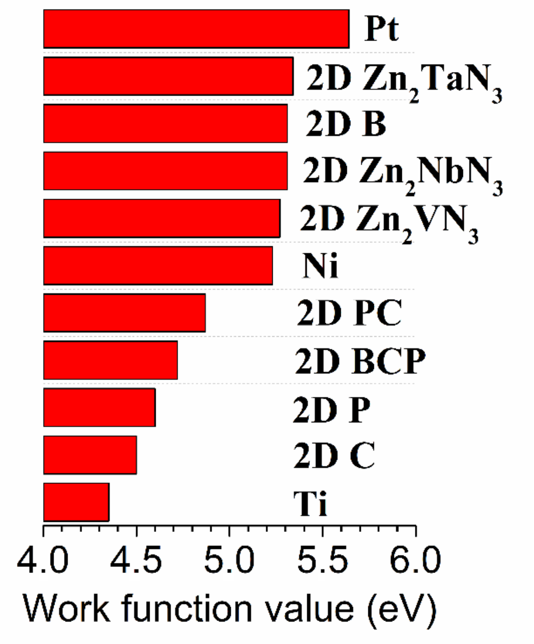

Figure S8. Work function of the Zn_2 (V, Nb, Ta)N_3 monolayer and other common 2D materials and bulk metals.

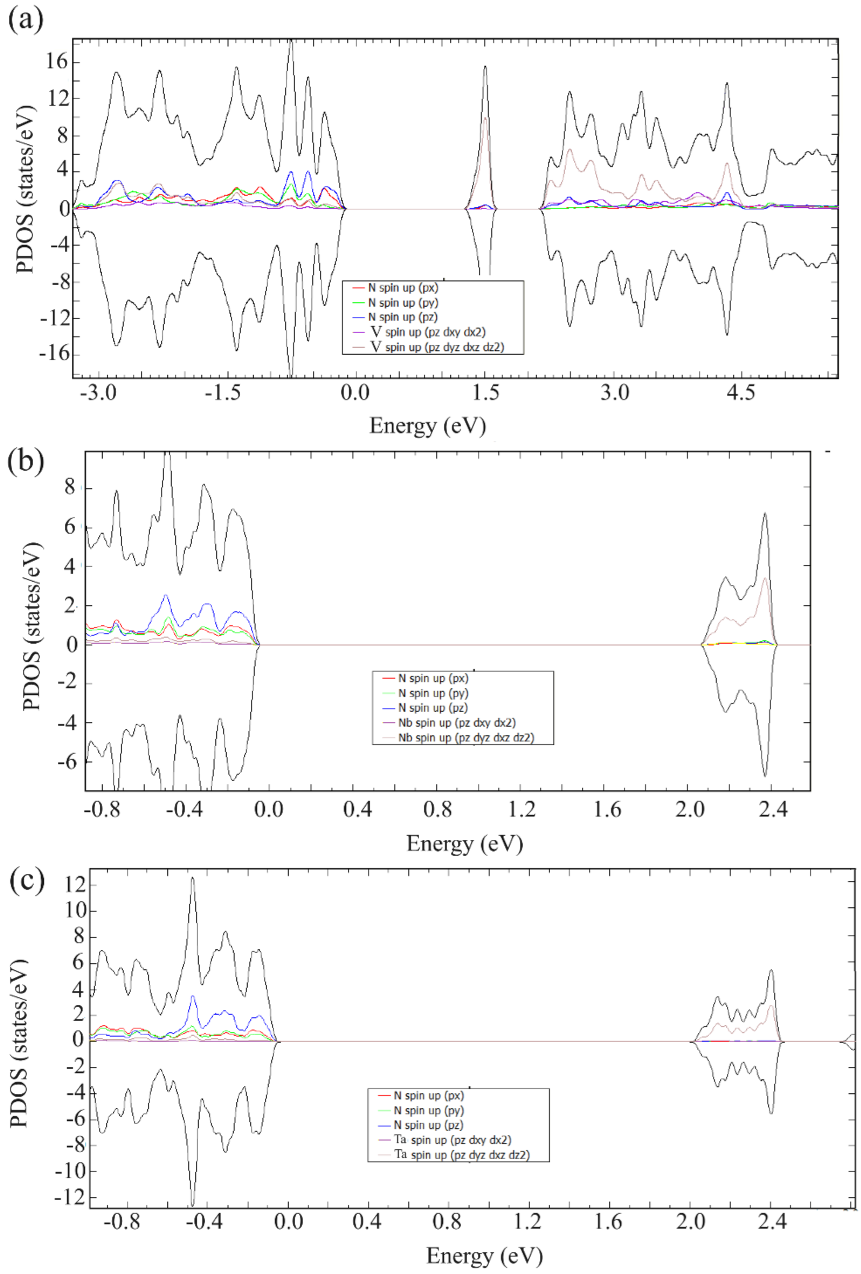

Figure S9. Orbital resolved DOS of the **(a)** Zn_2VN_3, **(b)** Zn_2NbN_3, and **(c)** Zn_2TaN_3 monolayers, obtained via the PBE GGA functional.

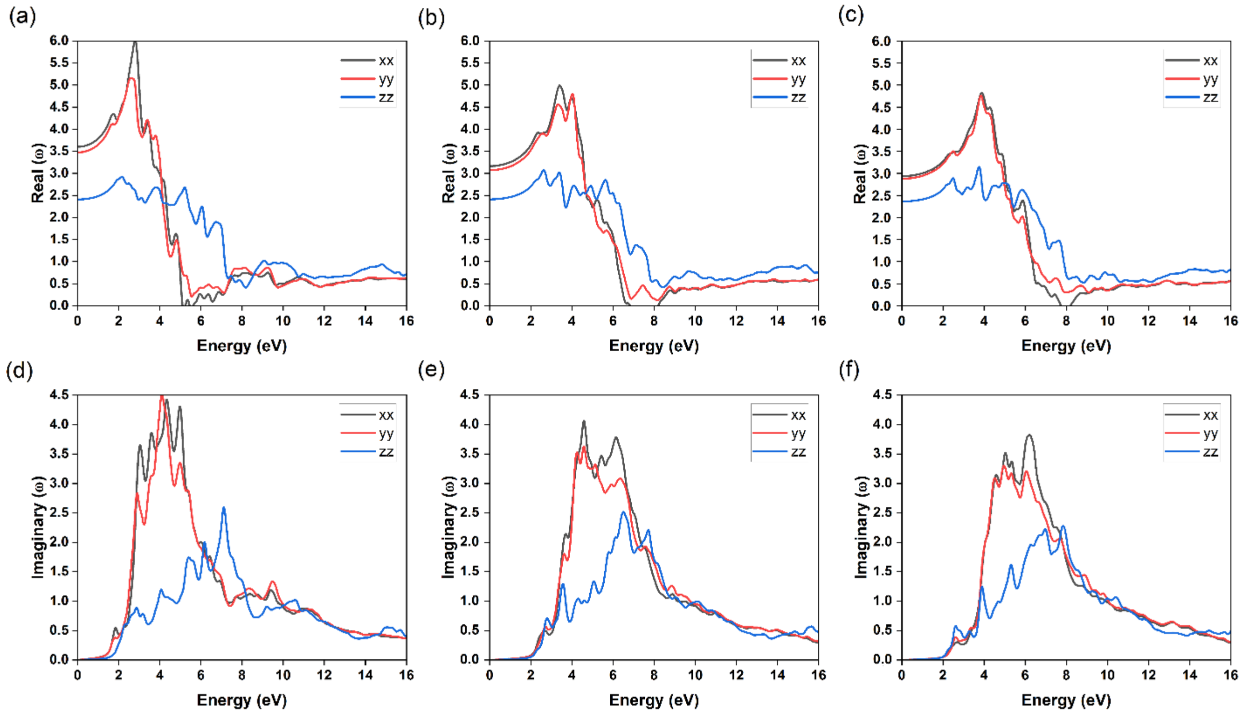

Figure S10. Real part of the dielectric function versus energy for the **(a)** Zn_2VN_3, **(b)** Zn_2NbN_3, and **(c)** Zn_2TaN_3 monolayers. Imaginary part of the dielectric function versus energy for the **(d)** Zn_2VN_3, **(e)** Zn_2NbN_3, and **(f)** Zn_2TaN_3 monolayers.
